# Public health round-up

**DOI:** 10.2471/BLT.25.010925

**Published:** 2025-09-01

**World Humanitarian Day**

Between January 2024 and August 2025, the World Health Organization (WHO) recorded 2450 attacks on health care in 21 countries, resulting in 2060 deaths and 2395 injuries. Of these, 1392 targeted health personnel through killing, injury, abduction, arrest or intimidation. On World Humanitarian Day, 19 August, the Global Health Cluster and partners urgently called for protection of humanitarian action and safeguarding those risking their lives to deliver health care in crises.

**Figure:**
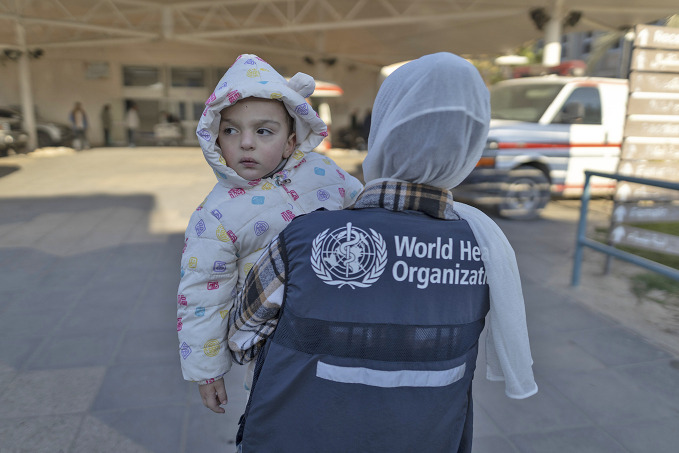
WHO

## Mental health support package

Over 300 million people worldwide now require humanitarian assistance due to conflict, climate change, political instability and disasters, creating an urgent demand for mental health support. To help meet this need, the World Health Organization (WHO) and partners have launched the *Mental health and psychosocial support minimum service package* , a tool designed to strengthen emergency mental health responses across humanitarian settings.

The tool outlines essential mental health and psychosocial support (MHPSS) activities, providing clear guidance for rapid, coordinated action. It includes step-by-step resources such as sector-specific quick start guides, training packages, a monitoring and evaluation indicator bank, and a global database of MHPSS training opportunities. 

Developed through an interagency collaboration led by WHO and the United Nations Children’s Fund (UNICEF), the tool equips humanitarian actors worldwide with practical, evidence-based guides to scale up life-saving mental health support.

“In emergencies, decisions need to be made quickly, and funding needs to be allocated, so that priority actions can be taken,” said Dévora Kestel, director a.i., Department of Noncommunicable Diseases and Mental Health at WHO. 

https://bit.ly/47DCZfB

## Health inequality monitoring

WHO has launched the Health Inequality Monitoring (HIM) Network, a global initiative to accelerate progress towards health equity. Established in June 2025 and hosted by WHO’s Department of Data, Digital Health, Analytics and AI, the network supports countries in monitoring and addressing health inequalities through capacity-building, evidence generation and the development of tools and best practices.

The HIM network’s founding members include 12 institutions across all WHO regions, selected from 68 applications received through an open call earlier this year. Members were selected on the basis of their training and capacity-building experience, technical capacity, engagement in national and global initiatives, publication and research contribution, as well as innovation and methodological development.

“The network has great potential to expand health inequality monitoring practices in Member States and promote its impact for advancing health equity,” said Ahmad Reza Hosseinpoor, lead of Health Equity Monitoring at WHO. “The institutions bring diverse skills, expertise and perspectives that will strengthen and enrich this area of work.”

The network will be guided by a Steering Committee and Secretariat at WHO, with a second call for new members planned in early 2026.

https://bit.ly/4fKVm4B

## Report on contaminated medicines 

A landmark report exposing the ongoing threat of contaminated medicines was released jointly by WHO and the United Nations Office on Drugs and Crime (UNODC). Over the past 90 years, at least 25 documented incidents of pharmaceutical excipient contamination have caused more than 1 300 deaths worldwide, with the majority occurring in low- and middle-income countries.

The report, *Contaminated medicines and integrity of the pharmaceutical excipients supply chain*, highlights how toxic industrial chemicals such as diethylene glycol and ethylene glycol have been illegally substituted for pharmaceutical-grade excipients such as propylene glycol, glycerin and sorbitol, often used in the formulation of medicines, including children’s cough syrups. Recent cases in the Gambia, Indonesia and Uzbekistan alone led to more than 330 child deaths.

Findings reveal systemic weaknesses in global supply chains, inadequate regulatory oversight, and deliberate criminal conduct, including falsification of excipients and documentation. The report calls for urgent global action to strengthen regulatory frameworks, enhance enforcement and improve cross-border cooperation to prevent further avoidable deaths.

https://bit.ly/4mSkzw4

## Triple elimination guidance

WHO has launched its first-ever guidance to support the triple elimination of mother-to-child transmission (EMTCT) of HIV, syphilis and hepatitis B virus. Presented at the 13th International AIDS Society conference on HIV Science in Kigali, Rwanda, in July 2025, the guidance aims to help countries design comprehensive, integrated programmes to prevent transmission of these infections during pregnancy, childbirth and breastfeeding.

The guidance, based on WHO’s Triple Elimination Framework, which promotes a person-centred, integrated approach to maternal and child health, provides governments, health-care providers and partners with strategies to assess, improve and expand EMTCT programmes, structured around four key pillars and four cross-cutting implementation considerations.

“The release of this new guidance marks a critical milestone in our collective efforts to end mother-to-child transmission of HIV, syphilis and hepatitis B virus,” said Meg Doherty, former director of WHO’s Global HIV, Hepatitis and Sexually Transmitted Infections Programmes. “It comes at a time when integrated approaches to maternal and child health are needed more than ever to ensure achievement of global targets by 2030 and safeguard the health of future generations.”

https://bit.ly/3JoCaxm

https://bit.ly/47HjUt5

## Respectful maternal and newborn care

Leading global health and development agencies have released the *Compendium on respectful maternal and newborn care*, the first global implementation guide to help countries eliminate mistreatment and promote dignity in maternity services. The publication, developed by WHO, the United Nations Population Fund (UNFPA), UNICEF and the United Nations (UN) Special Programme on Human Reproduction (HRP), translates principles of respectful care into concrete strategies for health systems.

Mistreatment during childbirth remains widespread. A 2019 study in four countries, supported by HRP and WHO, found that more than 40% of women experienced abuse or discrimination during labour, with many reporting non-consensual medical procedures. Over 75% of episiotomies and 60% of vaginal examinations were conducted without consent.

“Respectful care is not a luxury, it is a fundamental human right that shapes health outcomes and people’s experiences of care,” said Hedieh Mehrtash, technical officer and co-lead of the compendium.

The guide provides evidence, tools and actions for governments and health managers, from policy reform and health worker training to involving women and families in planning and monitoring services, helping ensure every woman, baby and family receives care grounded in dignity and respect.

https://bit.ly/4oCn47s

https://bit.ly/4mXIQ45

## Environmental scorecards

WHO has released the 2024 update of its health and environment country scorecards, evaluating how countries are addressing eight major environmental threats to health. These include air pollution; unsafe water, sanitation and hygiene; climate change; biodiversity loss; chemical and radiation exposure; occupational risks and environmental risks in health facilities.

WHO’s health and environment country scorecards serve as a valuable tool for guiding national action. They provide detailed data across the eight key areas linking environment, climate change and health policies, promoting cross-sectoral engagement, and helping governments prioritize evidence-based interventions.

“Tackling environmental risks isn’t optional. it’s a prescription for better health, stronger economies and a safer future. You can’t have healthy people on a sick planet,” said Maria Neira, director of WHO’s Department of Environment, Climate Change and Health.

This year’s edition introduces a new summary score, offering a concise snapshot of how environmental conditions affect health. Comprised of 25 indicators, the score enables countries to track progress at national, regional and global levels, highlighting trends in exposures, health impacts and policy implementation, as well as identifying critical data gaps.

While large disparities exist between countries, shaped in part by differing levels of economic resources, every country has an opportunity to strengthen efforts to reduce environmental health risks. 

About 25% of the global burden of disease is linked to environmental threats that are largely preventable. By addressing these environmental risk factors through stronger policies, cleaner technologies and sustainable practices, preventable illnesses and deaths can be significantly reduced. 

https://bit.ly/45LnOPd

**Cover photo**

A student at a school in Lebidoti, Brokopondo District, Suriname.

**Figure:**
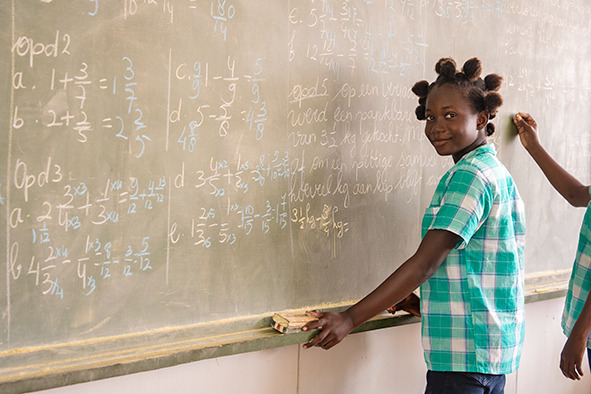
PAHO

**Looking ahead**

4 September. Implementing vision and hearing screening in schools, online event. https://bit.ly/3JrivwG

17 September. World Patient Safety Day: patient safety from the start, global events. https://bit.ly/4kPVpNb

25 September. Fourth UN High-level Meeting on the prevention and control of NCDs and the promotion of mental health and well-being. https://bit.ly/3JoxA2a

